# HPV Vaccination Attitudes and Behaviors among General Practitioners in Italy

**DOI:** 10.3390/vaccines9010063

**Published:** 2021-01-19

**Authors:** Francesco Napolitano, Concetta Paola Pelullo, Giorgia Della Polla, Italo Francesco Angelillo

**Affiliations:** 1 Department of Experimental Medicine, University of Campania “Luigi Vanvitelli”, Via Luciano Armanni, 5, 80138 Naples, Italy; francesco.napolitano2@unicampania.it (F.N.); concettapaola.pelullo@unicampania.it (C.P.P.); 2 Health Direction, Teaching Hospital of the University of Campania “Luigi Vanvitelli”, Via Santa Maria di Costantinopoli, 104, 80138 Naples, Italy; giorgia.dellapolla@unicampania.it

**Keywords:** attitudes, behaviors, GPs, HPV, Italy, vaccination

## Abstract

This cross-sectional electronic online or telephone survey assessed the attitudes and behaviors regarding human papillomavirus (HPV) vaccination and the effect of different factors among a nationally representative random sample of 349 general practitioners (GPs) in Italy. A semi-structured interview was performed between September 2018 and October 2020. Almost all respondents considered the HPV vaccine safe with an overall mean value of 8.8, on a scale ranging from 1 to 10, and 59.9% and 32.6% believed that the vaccination was very effective in preventing the related diseases among 12–26 years’ girls and boys. Multivariate logistic regression analysis showed that GPs who had received information about HPV vaccination from scientific journals were more likely to have positive attitude towards the effectiveness of the vaccine in preventing HPV-related diseases in girls between 12–26 years. A large majority (81.5%) of GPs who provided assistance to girls’ patients aged 11–12 years often or always recommend the HPV vaccine to them, and this behavior was more likely to occur in those who believed that the vaccine was very effective in preventing HPV-related diseases in girls between 12–26 years. GPs were more likely to often or always recommend the HPV vaccine to boys aged 11–12 years if they often or always recommended the vaccine to girls aged 11–12 years, if they believed that the vaccine was very effective in preventing HPV-related diseases in boys between 12–26 years, and if they considered the HPV vaccine very safe. GPs should receive information about the HPV immunization to ensure that they routinely communicate with their patient population in order to achieve better coverage rates.

## 1. Introduction

It is well-known that the human papillomavirus (HPV) infection is still one of the most serious public health problems in developed and developing countries, due to the fact that it is one of the leading sexually transmitted infections and is associated with genital warts, cervical, anogenital, and oropharyngeal cancers [1,2]. Although studies have suggested that the introduction of the HPV vaccination is associated with a decrease of the burden of the diseases [3,4], the uptake is lower than expected in several countries. In Italy, the vaccination is mainly provided in primary care practices and recommended for females and males aged 11 or 12 and for females through age 26 years and males through age 21 years if not previously vaccinated [5]. However, in 2018, only 40.3% of Italian girls and 20.8% of boys 12 years of age had completed the HPV vaccine series [6] and the coverage rates are still lag far below national goals [5]. Therefore, health-promoting advice and HPV prophylactic vaccination programs are no doubt one of the major areas to be acted upon. General practitioners (GPs) are called to play this role and they should be familiar with various aspects of HPV and be able to provide evidence-based advice to their patients. Recommendations from GPs to receive the vaccine, including addressing parents’ concerns, are an important factor in vaccinating their patients.

In spite of the important role that GPs have in HPV prevention, attitudes and behaviors towards HPV vaccination and their predictors among physicians who practice in several health care settings in different geographic areas have received attention in the literature [7,8,9,10], whereas similar studies have not been done among GPs in Italy. Therefore, the current national cross-sectional study attempts to fill this gap and the results might be important for the government to formulate the best approach to improve HPV vaccination coverage in the future. The primary objective of the current study was to assess the attitudes and practice regarding HPV vaccination among GPs and the second objective was to quantify the effect of different factors on GPs’ attitudes and commitment to vaccination in Italy.

## 2. Materials and Methods

### 2.1. Study Population

The survey lasted from September 2018 till October 2020 and participants were recruited by a nationwide two-stage clustering sample design. The sampling frame included all local health units (LHUs), which provide health care services to the population either directly, through their own facilities, or paying for the services provided by independent public and private structures. In the first stage, 19 LHUs were randomly selected. Initially, the research team contacted the head of each selected LHU with an e-mail explaining the purposes of the study, and asking for their assistance in obtaining names and contact information (e-mail address and phone number) of all GPs working in their LHU. In the second stage, a random sample of GPs was drawn from each LHU of the sample frame.

The sample size for this study was calculated using single population proportion formula with 95% confidence level, an expected proportion of 85% of GPs who often or always recommend the HPV vaccination to girls at 11 to 12 years, and relative precision to be 5%. In addition, since a 60% response rate was expected, the total number of enrollment was 327 GPs.

### 2.2. Procedures

Selected GPs received an invitation to participate through an e-mail asking to complete the survey. GPs were provided with a hyperlink to the online electronic survey located on a platform (Lime Survey project, Hamburg, Germany). The e-mail contained a cover letter with the lead investigator’s name and contact details, information about the background of the study, its goals and its voluntary nature, the importance of gaining their perspectives on the issue, the content of the questionnaire, the use and anonymization of the data, and the maximum time needed to complete the questionnaire (10 min). Links were unique to each participant, thereby eliminating the potential for multiple responses being provided by the same individual. Each link was collected and stored independently of survey responses to guarantee the anonymity and to know each participant. A brief introduction of study purpose and participants’ right was showed at the beginning of the questionnaire and participants were also informed that they provided their consent by participating. Participants were also informed that they were free to interrupt the interview without justification at any point during the survey and that when sending back their questionnaires, they were not able to withdraw the data. To maximize the survey response rate, e-mail reminders were sent at seven-day intervals to the GPs if there was no response and subsequently, three follow-up calls were made in different time slots during weekdays and weekends. In the follow-up calls, GPs were asked if they would prefer to participate by responding in a one-to-one telephone interview. If the email of the GP failed to deliver or the phone call was not available, an alternative GP was randomly selected instead from the same LHU. The participants were not compensated with financial or other incentives for their time spent.

### 2.3. Instrument

In a pilot study, the questionnaire was pretested on 25 GPs, and the results were used in order to verify ease of completion and refine the wording of the question, which were excluded afterward from the survey. The instrument, guided by previous published epidemiological surveys conducted by some of us [10,11,12,13], was structured in fourth sections. The survey began with questions about personal (age, gender, marital status) and professional characteristics (year of graduation, specialty, practice location, number of years in practice, practice size, number of their patient population in different target age groups). The second section asked questions about attitude toward HPV vaccination such as the effectiveness in preventing the related diseases among male and female between 12–26 years and if the HPV vaccine was considered to be safe. The third section asked questions if they routinely collect information on the sexual habits, recommended the HPV vaccination, administered the vaccine or referred patients for vaccination, and verified whether the vaccine had been administered, as well the reasons of the parents for refusing the vaccination for their daughters and sons. The last section consisted of questions about sources of information related to HPV infection and vaccination and whether they would like to get more information. The survey included open-ended responses, categorical responses, such as yes/no/do not know, ten-point Likert-type scale, and selection of responses from a list either through drop-down menus that allowed single responses or check boxes that allowed multiple responses. Participants were not able to continue to the next question if they failed to provide a response to an item. This ensured no data were missing.

### 2.4. Data Analysis

All statistical analyses were performed using Stata software v15 (StataCorp., College Station, TX, USA) [14]. First, descriptive statistics were used to analyze the results using counts and proportions for categorical data and means and standard deviations for continuous variables. In the second step, Chi-square test or Student’s *t*-test, as appropriate, explored the relationship between the different outcome variables and the selected variables. A *p*-value of 0.25 or less at bivariate analysis was considered for variables to be subsequently included in multivariate logistic regression models. In the third step, multivariate logistic regression models for dichotomous outcomes were used to estimate the association between the different covariates and the following outcomes of interest: positive attitude about the effectiveness of vaccine in preventing HPV-related diseases in girls between 12–26 years (Model 1), profile of GPs who often or always recommended the vaccine to girls aged 11–12 years (Model 2), and profile of GPs who often or always recommended the vaccine to boys aged 11–12 years (Model 3). The following covariates were included in the multivariate logistic models: age (continuous), gender (male = 0; female = 1), number of years since degree (continuous), number of years in practice (continuous), number of hours worked per week (continuous), source of information related to HPV infection and vaccination (none or no scientific journals = 0; scientific journals = 1), and need of additional information about HPV vaccination (no = 0; yes = 1). Moreover, the following variables were also included: positive attitude about effectiveness of HPV vaccine in preventing HPV-related diseases among 12–26 years’ girls (no = 0; yes = 1), and positive attitude about effectiveness of HPV vaccine in preventing HPV-related diseases among 12–26 years’ boys (no = 0; yes = 1) in Models 2 and 3, and often or always recommend the vaccine to girls aged 11–12 years (no = 0; yes = 1) in Model 3.

Backward stepwise selection of variables was performed to determine the final models. Odds ratios (ORs) and 95% confidence intervals (95% CIs) were calculated to measure the strength of the association between explanatory and outcome variables. All *p*-value were two-sided and the value below or equal 0.05 was considered to be statistically significant.

## 3. Results

### 3.1. Socio-Demographic and Professional Characteristics

Of the 567 GPs who were selected, 349 completed the interview for a response rate of 61.5%. The survey population’s demographic and professional characteristics are summarized in Table 1. More than two-thirds were males, the mean age was 59.3, they are working from a mean of 23.8 years, one-third worked for 34 h per week, and they had 11.3 and 9.7 girls’ and boys’ patients aged 11 or 12 years, respectively.

### 3.2. Attitudes

Regarding the participants’ attitudes, almost two-thirds (59.9%) and one-third (32.6%) exhibited the higher scores on a ten-point Likert-type concerning the effectiveness of the HPV vaccination in preventing the related diseases among 12–26 years’ girls, with a mean value of 9.2, and boys, with a mean value of 8.1. Moreover, almost all (97.1%) considered the HPV vaccine safe with an overall mean value of 8.8, on a scale ranging from 1 to 10. To avoid potential confounders, multivariate logistic regression analysis was performed in order to assess which independent variable would be significantly related to the different outcomes of interest and the results are reported in Table 2. The results revealed that those participants who had received information about HPV vaccination from scientific journals were almost 2.5 times more prone to have positive attitude towards the effectiveness of the vaccine in preventing HPV-related diseases in girls between 12–26 years compared to those who did not use this source (95% CI = 1.56–3.86) (Model 1).

### 3.3. Behaviors

Table 3 reported the behaviors of the GPs respondents towards HPV vaccination. Only less than one third of the respondents stated that they often or always collect information on the sexual habits of 12–26 years’ girls (32.5%) and boys (27.7%). Regarding the HPV vaccination behaviors, a large majority (81.5%) of GPs who provide assistance to girls’ patients aged 11–12 years often or always recommend the HPV vaccine to them, mainly because the vaccine is safe (71.9%), effective (69.3%), and that it prevents HPV-related diseases (33.1%). The results of the multivariate logistic regression analysis showed that GPs who believed that the vaccine was very effective in preventing HPV-related diseases in girls between 12–26 years (OR = 2.1; 95% CI = 1.15–3.89) were more likely to often or always recommend the HPV vaccine to girls aged 11–12 years (Model 2 in Table 2). Half of the participants (50.5%) reported that parents refused the vaccination for their daughters aged 11–12 years, and when asked why they refused, 80.8% indicated that they had concerns about vaccines for the potential side effects, followed by the lack of confidence about HPV vaccine efficacy (20.9%) and an inadequate knowledge of the infection (18.1%). Only 7.9% of GPs always verify that girls aged 13–26 years have been vaccinated against HPV, while 2.7% and 13.4% of them never or rarely do it, respectively. Two-thirds of GPs (66.2%) often or always recommend the HPV vaccine to girls aged 13–26 years and more than half (55.4%) reported that the main reason for refusing the vaccination among daughters was due to the parents’ concern regarding the side effects (66.9%), followed by beliefs that HPV infection was not serious (30.6%), and inadequate knowledge of the infection (30.5%).

Among GPs who provide assistance to boys’ patients aged 11–12 years, only one-third (37.7%) often or always recommend the vaccine and 19.3% never do it. The results of the multivariate logistic regression analysis showed that three variables were significantly associated with an increased probability to often or always recommend the HPV vaccine to boys aged 11–12 years. The variable GPs often or always recommending the vaccine to girls aged 11–12 years was found to be the strongest predictors for often or always recommending the HPV vaccine to boys aged 11–12 years since respondents that reported recommending the vaccine to these age-group girls were more than 6 times more likely to recommend the vaccination to boys of the same age (OR = 6.76; 95% CI = 2.57–17.78). Additionally, GPs who believed that the vaccine was very effective in preventing HPV-related diseases in boys between 12–26 years (OR = 1.99; 95% CI = 1.14–3.5) and those who considered very safe the vaccine (OR = 2.03; 95% CI = 1.19–3.46) were also more likely to often or always recommend the HPV vaccine to boys aged 11–12 years (Model 3 in Table 2).

Among those GPs who indicated that they recommend the vaccine, the main reasons were that the vaccine was effective (68.7%), safe (68.1%), and that it prevents HPV-related diseases (21.9%); whereas, the main reasons for not recommending were the lack of information (40.4%), concerns about the efficacy (21.3%), and the safety (17.9%). More than two-thirds of GPs (68.6%) reported that parents refused to vaccinate their sons and the main reasons were concern regarding the side effects (63.9%), lack of knowledge of the infection (30.4%), and because they felt that their boys were not at risk (21.1%). Moreover, only 17.7% of GPs verified that boys aged 13–26 years have been vaccinated and only 12.7% often or always recommended the vaccine to boys not previously vaccinated.

### 3.4. Sources of Information

The large majority of participants had received information about HPV infection (81.4%) and the related vaccination (73%). The most preferred sources where the GPs got the information about HPV infection and vaccination were scientific journals (71.7%), followed by scientific meetings (42.5%), colleagues (19.2%), and the internet (13.1%). More than two-thirds (68.8%) of participants were willing to acquire more information related to HPV infection and vaccination.

## 4. Discussion

This cross-sectional survey aimed to describe the attitudes and behaviors with regard to HPV vaccination among GPs in Italy as one of the priority groups for health-promoting advice and the prophylactic vaccination program. In addition, the study assessed the relationships of several factors with the GPs’ attitudes and decision to vaccinate against HPV.

In this study, the majority of the GPs respondents showed a very positive attitude regarding the HPV vaccination in general concerning, for example, the fact that it was considered to be safe and to be effective in preventing the related diseases among 12–26 years’ girls and boys by the vast majority of respondents. The important role of these attitudes was corroborated by the fact that those GPs who considered the vaccine very safe were more likely to often or always recommend the HPV vaccine to boys aged 11–12 years. However, in this study, despite demonstrating this mostly favorable opinion of the respondents regarding the HPV vaccination, one area was detected that may be improved among GPs with continued education. Indeed, unfortunately, it has been observed that the proportion of GPs who often or always recommended the vaccine to the boys’ patients aged 11–12 years was very low with a value of 37.7%. Therefore, targeted health promotion and prevention interventions are needed and must be implemented directed to change this practice in order to reach the target of 95% coverage for this age group. Moreover, based on the multivariate logistic regression model which examined the impact of different predictors on HPV vaccine recommendations, it was found that GPs that recommend the vaccination to girls aged 11–12 years were more likely to recommend often or always the HPV vaccine to boys of the same age. Of interest, the findings from this survey provide further insights into the reasons for not recommending the HPV vaccination to boys. It was surprising that the major impediments to HPV vaccination recommendation reported by respondents were the lack of information, negative perceptions about the efficacy and the safety of the vaccine. While an overwhelming concern in terms of perceived vaccine safety and efficacy was dominant in this survey for the parents for refusing the HPV vaccination for their sons, parents indicated also the lack of knowledge of the infection, and because they did not feel that their sons were at risk. These findings were also consistent with the results from similar previously studies [14,15,16,17,18,19]. The poor GPs and parents’ knowledge and attitudes on the important role of the vaccination will prevent them from being proactive respectively in recommending and requesting vaccination services. These findings suggest that GPs would benefit from ongoing education and vaccination improvement strategies because communication between GPs and their patients could result in more effective implementation of the HPV vaccination within the clinical practice setting. Information and communication campaigns are considered very important strategies globally to improve parents’ awareness and knowledge of vaccination, also emphasizing vaccine safety and efficacy. These campaigns are strongly needed for reducing vaccine hesitancy and refusal and for increasing immunization coverage and improving disease control. This is also confirmed by the finding that those GPs who believed that the vaccine was very effective in preventing HPV-related diseases in girls and boys between 12–26 years were more likely to often or always recommend the HPV vaccine to them. A striking finding of this study was that less than one third of GPs interviewees stated that they often or always collect information on the sexual habits of 12–26 years’ girls and boys. It is worth mentioning that GPs should be aware of population groups at risk for unhealthy sex life habit since such behavior plays an important role in the process of high-risk HPV persistent infection for adolescents and young adults [20,21,22]. Therefore, it is important to know the adolescent’s sexual habits in order to prioritize preventive strategies and to develop educational campaigns that should be routinely conducted to prevent the spread of HPV.

Sources of information are necessary to gain more HPV-related knowledge. However, one of the most disturbing and surprising findings of the study was that more than one-fourth of the surveyed GPs did not have received information about the HPV immunization. This underscores that it is essential that GPs receive additional information for ensuring that they routinely recommend the vaccine and for the communication dissemination to patients. The important finding that more than half of GPs wanted to know more information related to HPV infection and vaccination supports this. Results of the current study indicated that scientific journals were the most common source of information being identified by the GPs. It is important to highlight that scientific journals have a key and essential role in providing information. Indeed, when it has been measured with multivariate logistic regression analysis the relationship between the sources of information and the different outcomes of interest, it has been found that this source appears to have a significant influential impact on vaccination coverage since those who sought information from scientific journals were more prone to have positive attitude towards the effectiveness of vaccine in preventing HPV-related diseases in girls between 12–26 years compared to those who had not used such source. This result corroborated evidence that has already been proven in a lot of previous published investigations among different groups of the population that these sources are key in improving the level of knowledge, the attitudes, and the recommendation behaviors [10,23,24,25]. In addition, this is also important because increases in misinformation about vaccines may also limit or influence the acceptance, and with the pandemic emergency of COVID-19, there has been the shifting of healthcare resources with the accumulation of susceptible individuals and a higher likelihood of vaccine-preventable diseases outbreaks. Furthermore, it should be pointed out that the evaluation of vaccination depends on used methods, therefore, it is important to refer to the detection and quantification of corresponding pharmaceuticals [26].

As with all similar research, certain limitations of this study should be put into consideration when interpreting the findings and results for the development of broad-scale policy framework. First, the cross-sectional questionnaire-based study design has its own limitations and, therefore, it might be difficulty to draw causative links between the independent variables and the outcomes of interest. Second, the use of the questionnaire with self-reported information may constitute a bias as GPs could be under-reporting or over-reporting their behaviors. Third, the self-reporting nature does not allow for independent validation of the respondents’ answers, especially with regards to HPV behaviors and is also vulnerable to social desirability bias, thus it is possible that respondents may not report truthfully and they tend to answer questions in a manner that will be favorably viewed by others. However, potential bias may have been partially minimized because it has been conducted as an anonymous survey and all GPs were assured that their answers were confidential. Fourth, it is also possible that GPs who may have had an interest in HPV vaccination or who recommend the vaccine were more likely to respond than those who did not share these attitudes and practices. Despite these limitations, the current findings will provide an update about the attitudes and behaviors regarding HPV vaccination in Italy and have important implications for future interventions.

## 5. Conclusions

In conclusion, importantly, the information acquired from this nationally representative sample of GPs has implications for different policy designs and provides public health perspectives on several fronts for HPV vaccination. First, the positive attitude concerning the effectiveness of the vaccination in preventing the related diseases between girls and boys 12–26 years might result in a higher recommendation of the vaccine mainly among boys. Second, given the low proportion of GPs who often or always recommended the vaccine to the boys aged 11–12 years, it is important to monitor such rate in order to reduce the burden of HPV-related diseases. Finally, GPs should receive information about the HPV immunization for ensuring that they routinely communicate with their patient population for addressing myths regarding the vaccination for increasing adherence rates.

## Figures and Tables

**Table 1:** Socio-demographic and professional characteristics of the study population.

Characteristics	*N*	%
Gender		
Male	252	72.2
Female	97	27.8
Age, years	59.3 ± 7.3 (36–70) *	
Children		
Yes	280	83.1
No	57	16.9
Number of years since degree	32 ± 8.1 (1–45) *	
Number of years in practice	23.8 ± 11.1 (1–44) *	
Number of hours worked per week	33.8 ± 10.3 (5–90) *	
Number of 11–12 years old girl patients	11.3 ± 9.2 (0–70) *	
Number of 13–26 years old girl patients	72.5 ± 43.6 (0–213) *	
Number of 11–12 years old boy patients	9.7 ± 9.7 (0–70) *	
Number of 13–26 years old boy patients	71.7 ± 42.3 (2–200) *	

* Mean ± standard deviation (range).

**Table 2:** Results of multivariate logistic regression analysis to characterize the factors associated with the different outcomes of interest.

	OR	SE	95% CI	*p*-Value
**Model 1.** Positive attitude about the effectiveness of vaccine in preventing human papillomavirus (HPV)-related diseases in girls aged 12–26 years				
Log likelihood = −225.2, χ^2^ = 18.65 (3 df), *p* = 0.0003				
General Practitioners (GPs) who received information about the vaccine from scientific journals	2.45	0.57	1.56–3.86	<0.001
GPs who did not need additional information about the HPV vaccination	0.73	0.18	0.45–1.19	0.21
Younger GPs	0.98	0.01	0.95–1.01	0.27
**Model 2.** GPs who often or always recommended the vaccine to girls aged 11–12 years				
Log likelihood = −148.66, χ^2^ = 13.18 (3 df), *p* = 0.0043				
GPs who believed very effective the vaccine in preventing HPV-related diseases in girls aged 12–26 years	2.1	0.66	1.15–3.89	0.016
GPs who worked a lower number of hours per week	0.98	0.01	0.95–1.01	0.057
GPs who did not need additional information about the HPV vaccination	0.43	0.15	0.22–0.84	0.09
**Model 3.** GPs who often or always recommended the vaccine to boys aged 11–12 years				
Log likelihood = −174.45, χ^2^ = 51.36 (5 df), *p* < 0.0001				
GPs who often or always recommended the vaccine to girls aged 11–12 years	6.76	3.33	2.57–17.78	<0.001
GPs who believed that the vaccine was very effective in preventing HPV-related diseases in boys between 12–26 years	1.99	0.57	1.14–3.5	0.002
GPs who considered very safe the HPV vaccine	2.03	0.55	1.19–3.46	0.009
GPs who did not need additional information about the HPV vaccination	0.58	0.17	0.34–1.01	0.052
Older GPs	1.03	0.02	0.99–1.07	0.07

**Table 3:** Behaviors of the General Practitioners GPs respondents towards human papillomavirus (HPV) vaccination.

	*N*	%
Collecting information on the sexual habits of girls aged 12–26 years		
Often/always	103	32.5
Never/rarely/sometimes	214	67.5
Collecting information on the sexual habits of boys aged 12–26 years		
Often/always	84	27.7
Never/rarely/sometimes	220	72.3
Recommending the vaccine to girls aged 11–12 years		
Often/always	265	81.5
Never/rarely/sometimes	60	18.5
Recommending the vaccine to boys aged 11–12 years		
Often/always	115	37.7
Never/rarely/sometimes	190	62.3
Verifying that girls aged 13–26 years have been vaccinated against HPV		
Often/always	26	7.9
Never/rarely/sometimes	303	92.1
Verifying that boys aged 13–26 years have been vaccinated against HPV		
Often/always	54	17.7
Never/rarely/sometimes	251	82.3
Recommending the vaccine to girls aged 13–26 years not previously immunized		
Often/always	206	66.2
Never/rarely/sometimes	105	33.8
Recommending the vaccine to boys aged 13–26 years not previously immunized		
Often/always	35	12.7
Never/rarely/sometimes	240	87.3

Numbers for each item may not add up to total number of study population due to missing values.

## Data Availability

The data presented are available on request from the corresponding author.
